# Reproductive Toxicity and Multi/Transgenerational Effects of Emerging Pollutants on *C. elegans*

**DOI:** 10.3390/toxics12110785

**Published:** 2024-10-29

**Authors:** Zhiling Wu, Lingqiao Wang, Weihua Chen, Yiqi Wang, Ke Cui, Weiyan Chen, Jijun Liu, Huidong Jin, Ziyuan Zhou

**Affiliations:** 1 Department of Environmental Health, College of Preventive Medicine, Army Medical University (Third Military Medical University), Chongqing 400038, China; wzl2023@tmmu.edu.cn (Z.W.); mamababa520qq@163.com (L.W.); wangyiqi@tmmu.edu.cn (Y.W.); cuike777777@163.com (K.C.); weiyanchen@tmmu.edu.cn (W.C.); 17640061992@163.com (H.J.); 2 Central & Southern China Municipal Engineering Design and Research Institute Co., Ltd., Wuhan 430010, China; cwh361028@126.com; 3 Chongqing Center for Disease Control and Prevention, Chongqing 400707, China; cqliujijun2007@163.com

**Keywords:** emerging pollutant, reproductive toxicity, parental exposure, successive exposure, *C. elegans*

## Abstract

Emerging pollutants (EPs) are receiving increasing attention due to the threats they pose to the environment and human health. As EPs continue to emerge, risk assessment requires many model animals. *Caenorhabditis elegans* (*C. elegans*) has been an outstanding toxicological model organism due to its growth and development characteristics. Particularly, in studying the transgenerational influences of EPs, *C. elegans* has advantages in saving time and cost due to its short generation cycle. As infertility has become a major problem in human reproductive health, reproductive toxicities of EPs on contemporary nematodes and across generations of *C. elegans* were introduced in this review. Moreover, the underlying mechanisms involved in germ cell apoptosis, spermatogenesis, and epigenetic alteration were discussed. Future research opportunities and challenges are also discussed to expand our understanding of the reproductive influences of EPs.

## 1. Introduction

Infertility has become a global health issue [1]. There are many factors contributing to infertility, such as age, lifestyle, and environmental factors [2,3]. One of the major causes of increasing infertility is high exposure to environmental pollutants, which are pervasive and ensue via numerous routes [4]. Unlike conventional pollutants, emerging pollutants (EPs) refer to newly discovered or concerned pollutants that pose risks to the ecological environment or human health, with characteristics such as biological toxicity, environmental persistence, and bioaccumulation [5]. EPs include many chemicals used in daily life [6]. In China, the dominant types include perfluorinated compounds (PFCs), endocrine disruptors, microplastics (MPs), and antibiotics [7]. EPs can widely exist in water, soil, dust, air, and living organisms. As a result of their entry into the human body, they can cause adverse health effects, including organ injury, neurological damage, reproductive disorder, developmental disturbance, etc. To understand the EP-related reproductive disorders and fertility threat, a lot of research has been conducted [1,8]. Human exposure to EPs can have adverse effects on reproduction. For example, a study of a Chinese adult male population suggests that exposure to high doses of perfluoroalkyl and polyfluoroalkyl substances (PFAS) may be harmful to men’s reproductive health. Compared to serum PFAS, semen PFAS might serve as a more accurate indicator for men’s reproductive system [9]. Another Canadian population study showed a negative correlation between hair 2,2′,4,4′-tetrabromodiphenyl ether (BDE-47) concentration and sperm motility [10]. However, due to the limitations of research in humans, elucidating the molecular and biochemical mechanisms of human reproduction is challenging [11]. To gain a deeper view of the molecular and biochemical mechanisms of human reproduction, suitable animal models like rats and mice were selected for in vivo studies [12]. Prenatal exposure to Di-(2-ethylhexyl) phthalate (DEHP) had a significant effect on reproduction of male mice [13]. After exposure to perfluorooctanoic acid (PFOA) during pregnancy, fewer mice survived, their testicles were damaged, and their reproductive hormones were disrupted [14].

Although, mammalian models remain the gold standard for toxicology experiments, considering that many new pollutants in the environment are constantly detected, millions of individuals would be sacrificed in animal experiments. In addition to animal ethics, lots of labor, time, and materials would be consumed. A small, nonparasitic nematode, *Caenorhabditis elegans* (*C. elegans*) has been applied for studying many human diseases and has been one of the optimal animal models ever used. Based on these advantages, it is an extremely fascinating experimental model [15,16]: small size, short life span, high reproductive rate, and self-fertilize ability, which make it easy to obtain a mass of experimental worms. As the worm is transparent and easy to grow, reporter gene fusions, protein expression, and cell morphology can be observed in vivo. Moreover, it is the first multicellular organism in the world to complete full gene sequencing [17], which makes it possible to study toxicological mechanisms from a genetic angle. From nematodes to humans, many of the critical genetic pathways that regulate reproductive function are conserved.

**Figure 1:**
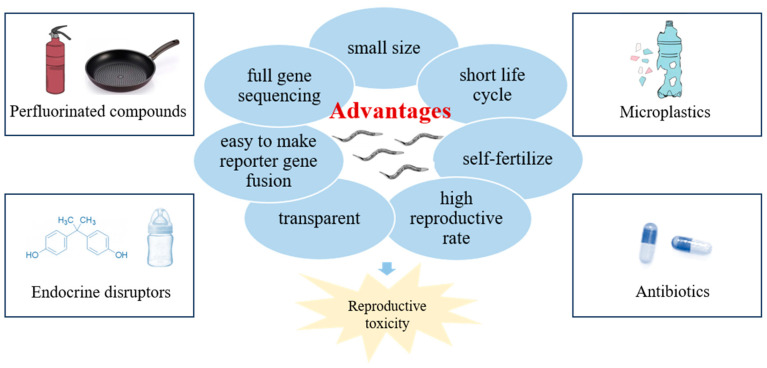
Advantages of the nematode model and the types of EPs discussed in this review.

This paper summarizes the recent progress of the reproductive effects of EPs using the model organism *C. elegans*. As shown in Figure 1, this article will mainly conduct a systematic discussion and put forward a prospect focusing on the reproductive toxicity induced by EPs, as represented by PFCs, endocrine disruptors, microplastics, and antibiotics. 

## 2. Methods

This review searched for articles related to the reproductive toxicity of EPs in *C. elegans* since 2018. Three major databases including Web of Science, Embase, and PubMed were searched with combinations of these search terms: “*Caenorhabditis elegans*”, “*C. elegans*”, “reproduction”, “reproductive”, “transgenerational”, “multigenerational”, “cross-generation”, “offspring”, “progeny”, “exposure”.

## 3. Reproductive Toxicity of EPs on Contemporary *C. elegans*

**Figure 2:**
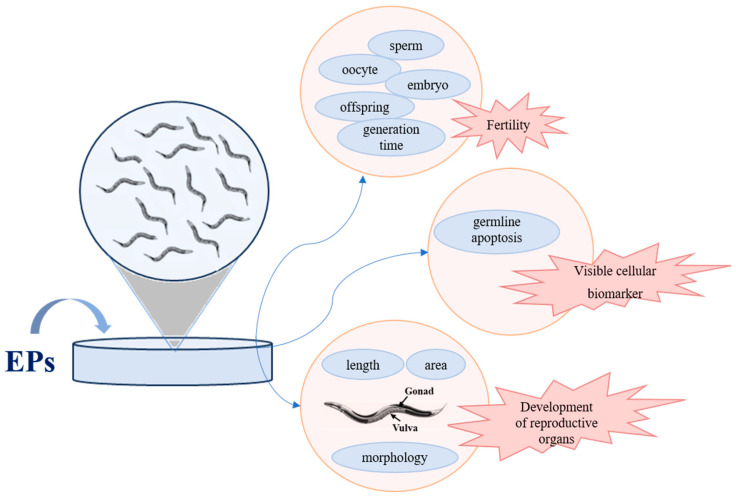
Assessment indicators of reproductive toxicity of *C. elegans*.

The reproductive toxicity of EPs on the worms was generally assessed from fertility, development of reproductive organs, and visible cellular biomarkers, which is shown in Figure 2. Table 1 summarizes the reproductive influence of the selected EPs. Visualized endpoints of reproductive toxicity were listed. As is shown, the brood size was selected as the endpoint in nearly all research listed to evaluate reproductive capacity. In addition to brood size, the number of oocytes, egg hatching, embryonic lethality, spermatid-related parameters, and generation time can also be used to evaluate fertility. Development of reproductive organs is another classification of endpoints, including the length of germline arm, gonadal area, and morphology of vulva. For the transparent body of *C. elegans*, germline apoptosis becomes visible cellular biomarkers to assess reproductive toxicity. Based on the findings, exposure to selected EPs caused reproductive damage.

### 3.1. PFCs

PFCs are synthetic alkane organic compounds, which have been widely used in many fields including food packaging materials, nonstick cookware, detergents, oil-proof treatment agents, and foam fire extinguishing agents [18].

Of the thousands of PFCs, PFOA and perfluorooctane sulfonate (PFOS) are the most studied. There were decreases in the number of germ cells, brood size, the size and motility of spermatid, and a rise in the rate of spermatid malformation observed following the exposure to PFOS and PFOA [19]. Reproductive toxicity effects were observed following perfluorobutane sulfonate (PFBS) and PFOS treatment in another study including significant reductions in brood number and egg production, and an increase in apoptosis in the gonads. By comparison, PFOS exhibits a greater capacity for bioaccumulation, while PFBS necessitates higher concentrations of exposure to induce reproductive toxicity [20]. However, the reproductive influences of additional PFCs have been less explored, including perfluorooctane sulfonamide (PFOSA), 6:2 chlorinated polyfluorinated ether sulfonate (F-53B), and perfluorohexanesulfonate (PFHxS).

### 3.2. Endocrine Disrupting Chemicals (EDCs)

EDCs, also known as environmental hormones or environmental estrogens, generally refer to exogenous chemicals to influence the endocrine system of organisms. There are a lot of EDCs and common examples including phenols, flame retardants, plastic additives, and pesticides. Previous studies have confirmed that EDCs could induce significant reproductive effects on *C. elegans*.

For instance, the brood size decrease was discovered under both nonylphenol (NP) and 4-nitrophenol (4-NP) exposure [21,22]. Abnormal reproductive systems, particularly irregular vulvas characterized by swelling, absence, and asymmetry, were also noted following exposure to NP. The overall count of germ cells was utilized to evaluate the impact on gonadal development toxicity under 4-NP exposure. Three studies researched the reproductive influences of di-(2-ethylhexyl) phthalate (DEHP) using identical concentrations [23,24,25]. These investigations revealed that DEHP has the potential to diminish reproductive capacity. One research focused on brood sizes, generation time, oocyte numbers, and apoptotic cells [23]. Alongside aspects of reproductive capacity like brood size, the quantity of fertilized eggs, the ratio of egg laying, and the hatchability of eggs, another study also focused on the advancement of the reproductive system, including the relative area of the gonadal arm [24]. Zongur’s study only focused on the number of eggs [25]. Fosthiazate and fenitrothion (FNT) are organophosphorus insecticides, and both of them can induce brood size reduction [26,27]. Atrazine (ATR) (≥0.04 mg/L) and carbendazim (10 μg/L) significantly decreased the worm’s reproduction with reduction rates of 17.06–35.41% and 43.71% in brood size [28,29]. Another experimental research exhibited that compared with 2,4-dichlorophenoxyacetic acid (2,4-D), cypermethrin (CYP), chlorpyrifos (CPF), and mancozeb (MCZ), ATR was the pesticide that had the greatest impact on brood size with a decrease rate of nearly 30% at the highest concentrations [30]. Tri-n-butyl phosphate (TnBP) and triphenyl phosphate (TPHP) are organophosphate flame retardants, while tetrabromobisphenol A (TBBPA) and BDE-47 are brominated flame retardants. All of them could impair the fertility of *C. elegans.* For example, following exposure to TnBP, significant effects on the reproductive ability and gonadal development of *C. elegans* were noted, along with a marked rise in germline apoptosis [31]. Treatment with BDE-47 may lead to a notable reduction in offspring numbers and promote germ cell apoptosis [32].

### 3.3. MPs

Global plastic production has grown exponentially since the invention of plastics in the early 19th century. Plastic fragments smaller than 5 mm in diameter are often considered MPs. There are a lot of types of MPs, including polypropylene (PP), polyamide (PA), polyvinyl chloride (PVC), polyethylene (PE), and polystyrene (PS) [33]. As a synthetic organic polymer, MPs possess traits such as small particle size, lightweight, extensive specific surface area, strong hydrophobicity, variable density, etc., allowing them to remain stable in the environment for an extended period. Because of the ubiquity and persistence of MPs, their toxic effects on organisms are of increasing concern.

PS is among the plastics that are frequently identified in the environment. Nano-sized PS soil exposure resulted in a decrease in eggs’ quantity presented in utero and the percentage of hatching, with reduction rates of 7–33% and 2.6–4.4%, and increase rates of 12–55% in the incidence of apoptotic germ cells within the gonads [34]. Media and particle size can affect reproductive toxicity. Research indicated that *C. elegans* responded more sensitively to the number of progeny in soil medium than in liquid medium. Additionally, in soil environments, *C. elegans* demonstrated increased sensitivity towards larger particles [35]. Chemical modifications to the surface can alter the physicochemical characteristics of MPs/nanoplastics (NPs) and influence their toxicity. Studies have indicated that amino modification groups may increase the toxicity of nanopolystyrene (NPS) on reproductive abilities and gonadal development [36]. Microplastics existing in the environment are susceptible to environmental factors (such as temperature, light, and mechanical stress), and undergo aging processes, which could lead to significant changes in the physicochemical properties and ecotoxicity of microplastics. Aged MPs were obtained by UV irradiation in two studies [37,38]. The reproductive effects of aged MPs were more severe compared to those of pristine MPs. MPs can absorb a large number of environmental pollutants due to their extensive surface area, so they can become transport carriers of environmental pollutants such as organic pollutants. Therefore, combinational effects between MPs and other contaminants were conducted. Research [39] demonstrated that NPS could significantly increase the toxicity of Microcystins-LR (MC-LR), leading to a decrease in brood size. However, the effect was affected by the concentration of NPS.

### 3.4. Antibiotics

Antibiotics have been used to treat disease and promote the growth of livestock. Antibiotics in the environment mainly come from the livestock and poultry industry, the aquaculture industry, and pharmaceutical wastewater. Antibiotics can directly influence the diversity of microbial communities, the growth and development of animals and plants, and human health. Recently, there have been limited studies relating to the effect of antibiotics on the reproductive toxicity of *C. elegans*. Huang et al. [40] surveyed exposing nematodes to Fluoroquinolone antibiotics (FQs), including enrofloxacin (ENR), ciprofloxacin (CIP), norfloxacin (NOR), ofloxacin (OFL), fleroxacin (FLE), lomefloxacin hydrochloride (LOM), and sarafloxacin hydrochloride (SAR). The result showed that all the FQ exposures could cause reproductive toxicity of *C. elegans*, resulting in a reduced brood size and lower egg hatchability. ENR exhibited the strongest inhibitory effect on reproduction among the seven FQs [40]. In addition, early-life long-term exposure to sulfamethoxazole (SMX) could also result in significant reproductive impairment [41].

**Table 1:** Summary of reproductive toxicity of EPs on *C. elegans*.

EPs		Exposure Condition	Visualized Endpoints of Reproductive Toxicity	Reference
PFCs	PFOS PFBS	0.1 μM (50.0 μg/L) 500, 1000, 1500 μM (150.0, 300.1, 450.15 mg/L)	egg production, brood size, apoptosis	[20]
	PFOS PFOA	0.001, 0.01, 0.1 mmol/L (0.5, 5.0, 50.0 mg/L) 0.001, 0.01, 0.1 mmol/L (0.4, 4.1, 41.4 mg/L)	brood size, spermatid (size, morphology, and activation), number of germ cells	[19]
EDCs	DEHP	0.1, 1, 10 mg/L	brood size, generation time, oocyte numbers, gonadal structure, apoptosis	[23]
	BDE-47	1, 3, 10, 30 µg/mL	offspring number, average time of egg-laying, apoptosis	[32]
	BPA	100, 500 μM (22.8, 114.1 mg/L)	egg number, brood size, embryonic lethality, apoptosis	[42]
	NP	1, 10, 200, 400 μg/L	brood size, gonad system	[22]
	Carbendazim	0.01, 0.1, 10, 100 μg/L	brood size	[29]
	ATR	0.0004, 0.004, 0.04, 0.4, 4, 40 mg/L	brood size, generation time	[28]
	TnBP	0.1, 1, 10, 100, 1000 μg/L	brood size, numbers of germline cells and fertilized eggs, area of gonad arm, apoptosis	[31]
	DEHP	0.1, 1, 10 mg/L	brood size, egg-laying rate, eggs in the uterus, gonadal area, germline cell numbers	[24]
	TCBPA	0.01, 0.1, 1, 10, 100 μg/L	brood size, apoptosis	[43]
	Fosthiazate	0.01, 0.1, 1, 10 mg/L	brood size	[26]
	ATR/2,4-D CPF/CYP/MCZ	1, 10, 100, 1000 µg/L 0.1, 1, 10, 100 µg/L	brood size, percentage of gravid nematodes	[30]
	FNT	0.4, 4, 40, 400 µg/L	brood size, germ cell numbers, generation time, gonadal development	[27]
	TPHP	1, 10, 100, 500 µg/L	germline apoptotic cells, number of embryos, total progeny per worm	[44]
	DEHP	0.625, 1.25, 2.5, 5, 10 mM (0.2, 0.5, 1.0, 2.0, 3.9 g/L)	number of eggs	[25]
	4-NP	8 ng/L, 8 µg/L	brood size, oosperm numbers, ovulation rate, total number of germ cells	[21]
MPs/NPs	Pristine/NPS-NH_2_	1, 10, 100, 1000 µg/L	brood size, number of fertilized eggs, length of gonad arm, relative area of gonad arm, germline apoptosis	[36]
	MC-LR NPS	0.1, 1, 10 µg/L 0.1, 1 µg/L	brood size	[39]
	PS	0.01, 0.1, 1, 10, 100 mg/L 0.01, 0.1, 1, 10, 100 mg/kg	number of offspring	[35]
	NPS	1, 10, 100, 1000 mg/kg	number of eggs in utero and hatched eggs, apoptosis	[34]
	Pristine/UV-PS	0.1, 1, 10, 100 µg/L	brood size, number of egg ejections, apoptosis	[37]
	Pristine/aged PLA-MPs	0.1, 1, 10, 100 µg/L	brood size, number of hatched eggs and fertilized eggs in the uterus, number of total germline cells, area and length of gonad arm, apoptosis	[38]
Antibiotics	ENR/CIP/NOR/OFL/FLE/LOM/SAR	5.56, 13.9, 27.8, 55.6, 139, 209, 278, 556 μmol/kg	brood size, egg hatchability, morphology of eggs in the uterus, apoptosis	[40]
	SMX	0.001, 1, 10, 100 mg/L	cumulative offspring	[41]

Abbreviations: PFOS: Perfluorooctane sulfonate; PFBS: Perfluorobutane sulfonate; PFOA: Perfluorooctanoic acid; DEHP: Di-(2-ethylhexyl) phthalate; BDE-47: 2,2′,4,4′-tetrabromodiphenyl ether; BPA: Bisphenol A; NP: Nonylphenol; ATR: Atrazine; TnBP: Tri-n-butyl phosphate; TCBPA: Tetrachlorobisphenol A; 2,4-D: 2,4-dichlorophenoxyacetic acid; CPF: Chlorpyrifos; CYP: Cypermethrin; MCZ: Mancozeb; FNT: Fenitrothion; TPHP: Triphenyl phosphate; 4-NP: 4-nitrophenol; NPS: Nanopolystyrene; NPS-NH_2_: Amino modified NPS; MC-LR: Microcystins-LR; PS: Polystyrene; UV-PS: UV-photodegraded PS; PLA-MPs: Polylactic acid MPs; ENR: Enrofloxacin; CIP: Ciprofloxacin; NOR: Norfloxacin; OFL: Ofloxacin; FLE: Fleroxacin; LOM: Lomefloxacin hydrochloride; SAR: Sarafloxacin hydrochloride; SMX: Sulfamethoxazole.

## 4. Multi/Trans-Generational Reproductive Toxicity of EPs on *C. elegans*

**Figure 3:**
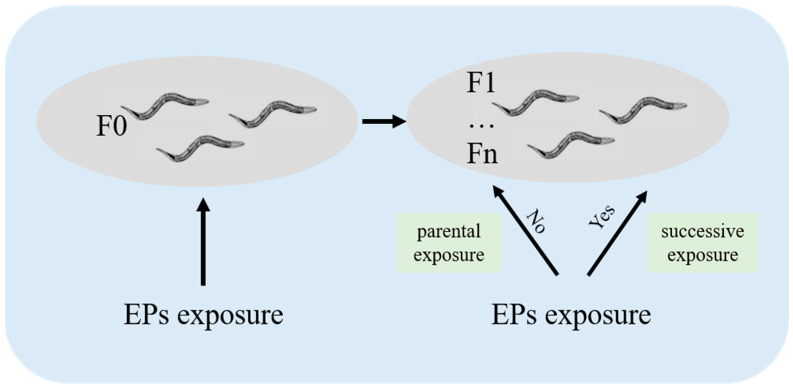
Diagram showing two types of exposure (F0, parent generation; Fn, the nth generation).

The generation time of *C. elegans* is 3–4 days, while the sexual maturity time of zebrafish and mice is approximately 3–4 months and 6–8 weeks, respectively [45,46,47]. Moreover, cultivating nematodes is both simple and inexpensive, allowing for the rapid collection of numerous samples [48,49]. Therefore, *C. elegans* have the advantage as model organisms to study multi/trans-generational toxicity. To confirm whether the toxic influences of EPs on the parent organisms would be passed on to their offspring, studies on the multi/trans-generational reproductive toxicity of EPs were carried out and are presented in Table 2. There are two categories of exposure in the assessment system for multi/trans-generational toxicity: parental exposure and successive exposure (Figure 3). Parental exposure refers to the scenario where only the parent generation (F0) is subjected to exposure, whereas successive exposure involves conducting exposure experiments on both the F0 nematodes and their subsequent offspring [50,51].

### 4.1. PFCs

Both parent and successive PFOS exposure were conducted in Chowdhury’s research. After PFOS parental exposure (0.01, 0.1, 1 μM), in all the P0 generations, significant reductions in brood size were found compared to the control, and in the later generations (F1–F5), the defects of brood size were recovered completely. Under conditions of exposure to PFOS over successive generations (0.001 μM), there was no notable variation in the overall number of offspring from P0 to F5 when compared to the control [52]. This may be associated with the low levels of PFOS; future studies could focus on higher concentrations of PFOS to examine the impact of ongoing exposure on reproductive toxicity in offspring.

### 4.2. EDCs

Prolonged exposure of parents to 200 nM of hexabromocyclododecane (HBCD) could greatly diminish the brood size of the F0 generation, but the brood size of the F1 generation did not change significantly, indicating that exposure of the F0 generation to HBCD would not have adverse reproductive effects on F1 generation [53]. However, under DEHP, tebuconazole (TEB), and tetrabromobisphenol A (TBBPA) exposure, reproductive toxicities could be caused in the parental generation, and these detrimental influences might also be detected in the offspring even in the absence of toxicant. Take DEHP as an example, after prolonged parental exposure, the total brood size among the F0–F4 generations significantly decreased when a light recovery was detected in the F5 generation [54]. The findings indicated that DEHP has the potential to influence the reproductive capabilities of the initial generations, yet the nematodes appeared to regain their functionality after experiencing multigenerational impacts. This recovery may be linked to the restoration of certain biological processes, including patterns of methylation. The results of parentally TEB exposure were similar to DEHP exposure when the reproduction defects gradually recovered starting from F3 [55]. Under exposure to TBBPA, reproductive toxicities were observed in both the G1 and G2 generations [56]. The brood size of G1 nematodes decreased significantly under TBBPA treatment of 10–1000 mg/L, while in G2 nematodes, the brood size decreased only in the highest treatment group, indicating that the reproductive toxicity of offspring recovered. Similar to PFOS exposure [52], bisphenol S (BPS) successive exposure (0.001 μM) had no significant influence on the brood size of G1 to G4 generations [57]. However, when the concentration increased (0.01 to 100 μM), the brood size across the four generations reduced significantly compared to the control. Both successive and parental exposure were conducted to identify the multi/transgenerational reproductive influence of Atrazine (ATR) [58]. Continuous exposure to ATR significantly increased the reproductive toxicity of the subsequent generations, and the damage to the F2 generation was most significant under parental ATR exposure.

### 4.3. MPs

Research showed that exposure to MPs/NPs could cause transgenerational reproductive toxicity, and the influences were affected by the plastic concentration, particle size, and the modification of the particles. For instance, exposure to PS-NPs at concentrations ranging from 1 to 100 μg/L during the P0 generation inhibited brood size, and reproduction defects were additionally noted in the offspring. However, the reproduction defects recovered starting from the F3, F4, and F5 generations, respectively [59]. PLA-MPs could also cause transgenerational reproduction inhibition [60]. To explore whether PS-NPs induced transgenerational reproductive toxicity was size-dependent, toxicity experiments exposure to 20 and 100 nm PS-NPs were conducted. The findings indicated that exposure to 20 nm particles led to more pronounced transgenerational toxicity compared to larger particles. The 20 nm PS-NPs exhibited reproductive toxicity in F1–F6 generations, whereas the 100 nm PS-NPs demonstrated toxic effects solely in the F1–F3 generations at identical concentrations [61]. As mentioned above, microplastic modification was another important factor affecting transgenerational toxicity. Pristine NPS was modified with amino groups, which were defined as NPS-NH_2_, and NPS-NH_2_ showed severe transgenerational reproductive toxicity [62]. In addition, compared with carboxyl-modified PS (PS-COOH), PS-NH_2_ still exhibited more severe transgenerational reproductive toxicity [63].

### 4.4. Antibiotics

Zhang’s group previously investigated the multi/trans-generational reproductive toxicity of several antibiotics [64,65,66]. For instance, sulfamethoxazole (SMZ) and erythromycin (ERY) were selected as the contaminants in Yu’s research. Both ERY and SMZ could inhibit reproduction across all generations, although the level of inhibition decreased over successive generations with ongoing exposure to ERY. In the case of SMZ, reduced inhibition was only noted in the F4 generation. Furthermore, even after complete cessation of exposure, ERY and SMZ continued to affect reproduction [66]. 

Until now, there have been relatively limited studies focusing on multi/transgenerational reproductive toxicity in *C. elegans*, and more attention should be focused on this aspect in the future.

**Table 2:** Summary of multi/trans-generational reproductive toxicity of EPs on *C. elegans*.

EPs		Exposure Condition	Exposure Method	Number of Generations	Visualized Endpoints	Reference
PFCs	PFOS	0.01, 0.1, 1 μM (5.0, 50.0, 500.13 μg/L) 0.001 μM (0.5 μg/L)	parental exposure successive exposure	six (P0–F5)	brood size	[52]
EDCs	DEHP	20 mg/L	parental exposure	six (F0–F5)	brood size	[54]
	HBCD	0.2, 2, 20, 200 nM (0.13, 1.3, 12.8, 128.3 μg/L)	parental exposure	two (F0–F1)	brood size	[53]
	BPS	0.001, 0.01, 0.1, 1, 10, 100 µM (0.3, 2.5, 25.0, 250.3, 2502.7, 25,027 μg/L)	successive exposure	four (G1–G4)	brood size	[57]
	TEB	0.01, 0.1, 1, 10 μg/L	parental exposure	five (P0–F4)	brood size, reproductive system abnormality, embryo hatchability	[55]
	TBBPA	0.1, 1, 10, 100, 1000 μg/L	parental exposure	two (G1–G2)	brood size	[56]
	Glufosinate	0.1, 1, 10, 100 μg/L	successive exposure	three (F0–F2)	brood size, number of pregnant eggs	[67]
	ATR	0.0004, 0.004, 0.04, 0.4, 4, 40 mg/L	successive and parental exposure	six (P0–F5)	brood size, fertilized eggs, oocytes, ovulation rate, bag of worms, germ cell numbers, relative area of gonad arm	[58]
MPs/NPs	PS-NPs	1, 10, 50, 100 mg/L/ 100 mg/L	successive exposure parental exposure	five (F0–F4)	brood size, germline apoptosis	[68]
	NPS/NPS-NH_2_	1, 10, 100 μg/L	parental exposure	five (P0–F4)	brood size, number of fertilized eggs and germline cells, length and area of gonad arm, apoptosis	[62]
	NPS	0.1, 1, 10, 100 μg/L	parental exposure	eight (P0–F7)	brood size	[61]
	NPS	1, 10, 100 μg/L	parental exposure	six (P0–F5)	brood size	[59]
	NPS-S	1, 10, 100 μg/L	parental exposure	five (P0–F4)	brood size, number of fertilized eggs	[69]
	PLA-MPs	1, 10, 100 μg/L	parental exposure	four (P0–F3)	brood size, number of fertilized eggs, gonad development, germline apoptosis	[60]
	PS/PS-NH_2_/PS-COOH	100 μg/L	parental exposure	five (P0–F4)	brood size, fertilized eggs, egg ejection rate, cell corpses per gonad	[63]
	NPS-NH_2_	0.1, 1, 10 μg/L	parental exposure	five (P0–F4)	brood size, number of fertilized eggs	[70]
Antibiotics	OFL NOR	2.6 ng/L 6.5 ng/L	successive and parental exposure	nine (F1–F9)	number of offspring	[64]
	ENR	229 ng/L	successive exposure	nine (F1–F9)	number of offspring	[65]
	ERY SMZ	0.022, 22.0 mg/L 0.036, 36.0 mg/L	successive and parental exposure	four (F1–F4)	number of offspring	[66]

Abbreviations: PFOS: Perfluorooctane sulfonate; DEHP: Di-(2-ethylhexyl) phthalate; HBCD: Hexabromocyclododecane; BPS: Bisphenol S; TEB: Tebuconazole; TBBPA: Tetrabromobisphenol A; ATR: Atrazine; PS: Polystyrene; PS-NPs: Polystyrene nanoplastics; NPS: Nanopolystyrene; NPS-NH_2_: Amino modified NPS; NPS-S: Sulfonate-modified NPS; PLA-MPs: Polylactic acid MPs; PS-NH_2_: Amino modified PS; PS-COOH: Carboxyl modified PS; OFL: Ofloxacin; NOR: Norfloxacin; ENR: Enrofloxacin; ERY: Erythromycin; SMZ: Sulfamethoxazole.

## 5. Underlying Mechanisms of Reproductive Toxicity Induced by EPs

Numerous mechanisms may function in the reproductive toxic influences of EPs on *C. elegans.* Potential mechanisms and associated genes are detailed in Table 3 and Table 4. As illustrated, the primary mechanisms are predominantly related to germ cell apoptosis, spermatogenesis, and epigenetic modifications. 

### 5.1. Apoptosis in Germ Cell 

The mechanism related to apoptosis is the most studied. Apoptosis represents a biologically conserved mechanism that is vital for the development and maintenance of homeostasis in the majority of multicellular organisms. Apoptosis usually occurs in the germ line of hermaphrodite nematodes and accounts for about 50% of developing oocytes [71]. As listed above, germ cell apoptosis is a sensitive endpoint for evaluating reproductive toxicity. In *C. elegans*, critical elements that contributed to the process of apoptosis comprise Apaf-1, along with Bcl-2 homologs CED-4 and CED-9, the BH3-only domain protein EGL-1, and the caspase CED-3 [72]. CED-4 dimers are sequestered through their contact with a single CED-9 molecule in living cells. EGL-1 associates with CED-9, prompting conformational alterations in CED-9 that ultimately disrupt the binding between CED-4 dimers and CED-9 in cells that are programmed for apoptosis. CED-4 dimers aggregate to form tetramers that subsequently engage with CED-3, facilitating its intrinsic activation upon dissociation from CED-9 [71]. Studies revealed that EPs could modulate the expression of genes related to apoptosis. Following exposure to TnBP, a reduction in the expression levels of the *ced-9* gene was observed, whereas the levels of the *ced-3* and *ced-4* genes exhibited an increase. This suggested that TnBP exposure had a significant impact on the CED-9-CED-4-CED-3 signaling pathway associated with apoptosis [31]. The signaling cascade could persist in the subsequent generations under the exposure of NPs [68]. Likewise, DEHP exposure upregulated *ced-3* and *ced-4* expressions and downregulated *ced-9* gene expression [23].

DNA damage can trigger germline apoptosis, and the mechanisms that trigger apoptosis are particularly complex. One of the most prominent examples is the worm protein CEP-1, which can enhance germline apoptosis caused by DNA damage through the modulation of EGL-1 activity [73,74,75]. DNA damage elevates the expression of *egl-1* (the proapoptotic gene), and the response relies on the involvement of *hus-1* and *cep-1* [73]. In nematodes, HUS-1, which is the ortholog of Hus1, functions as a checkpoint protein for DNA damage [73,74,75]. The *hus-1* gene regulates the expression of the *cep-1* gene [37]. To sum up, the HUS-1-CEP-1-EGL-1 signaling cascade is the upstream of the core apoptosis pathway to regulate the germline apoptosis. One research showed that apoptosis induced by TCBPA may be induced by DNA damage. The potential molecular mechanism appeared to be involved in DNA damage facilitated by *hus-1*, resulting in apoptosis through a *cep-1* dependent pathway [43].

The damage induced by oxidative stress is also significant in the reproductive toxicity of nematodes exposed to EPs [24]. Reactive oxygen species (ROS) are typically characterized as small, short-lived, and highly reactive molecules. Within cellular environments, the production of ROS is balanced by a diverse array of antioxidant defenses. These defenses encompass both enzymatic scavengers and non-enzymatic scavenging mechanisms [76,77]. When the antioxidant defense mechanisms are compromised, cells may suffer damage due to an overabundance of ROS, subsequently leading to the occurrence of apoptosis [77]. High concentrations of carbendazim exposure could cause severe oxidative stress, thus disrupting cell homeostasis and promoting cell apoptosis [29]. Another study further illustrated that germline apoptosis, triggered by oxidative stress, was a crucial factor in the reproductive toxicity caused by ENR [40]. BDE-47 has the potential to trigger oxidative stress, leading to germline apoptosis through a MAPK-mediated pathway that operates independently of p53 [32].

### 5.2. Spermatogenesis

Hermaphrodites and male nematodes both undergo spermatogenesis, which creates functional sperm from an undifferentiated germ cell. Spermatogenesis involves a sequence of critical events, encompassing mitotic and meiotic divisions, the transport of fibrous body-membranous organelles (FB-MO), and the activation of spermatozoa [19]. Research showed that some environmental pollutants may interfere with the expression of spermatogenesis-related genes so reproductive ability was affected [19,26]. Another study revealed that fenitrothion could impact the expression of androgen receptors via the modulation of cortisol and melatonin levels, thereby influencing androgen receptor signaling pathways that are critical for germ cell meiosis and sperm formation [27].

From current studies, mechanisms involved in spermatogenesis are still lacking and require further investigation.

### 5.3. Epigenetic Alteration

A single parental (F0) exposure to EPs can lead to alterations in reproductive toxicity, which are subsequently passed on to future generations. However, understanding of the transfer mechanism to subsequent generations is limited. Epigenetic inheritance is a potential mechanism for trans-generational effects. Epigenetics refers to heritable changes in gene function without changes to the DNA sequence [78]. Among the epigenetic modifications, DNA methylation is the most extensively researched and understood, followed by histone modifications and non-coding RNAs [79,80].

Under ATR exposure, transgenerational toxicity may arise from mitochondrial stress in the P0 generation, which was influenced by transcriptional 6 mA modifiers. Furthermore, these epigenetic marks could also be established in the offspring [58]. Reproductive toxic memory may be transmitted to subsequent generations via alterations in epigenetic modifications, such as 6-methyladenine (6mdA), which possess the potential for inheritance. MET-2 and SPR-5 collaboratively controlled histone methylation in the germline, and their deletion could cause transgenerational inheritance of H3K4me2, leading to offspring sterility [81,82]. Findings [68] also suggested that MET-2, SPR-5, and SET-2 were involved in NPs-induced transgenerational effects. Except for H3K4me2, H3K9me3 may also play an important role in regulating transgenerational toxicity [63].

## 6. Conclusions and Perspectives

In summary, this paper reviews the reproductive toxicity of EPs on *C. elegans* and discusses whether this toxicity can be transmitted to the next generation or subsequent generations. The potential mechanisms were also discussed. Studies based on nematodes as model organisms can provide references for investigating the detrimental impacts of EPs on other organisms or humans. However, to further understand the reproductive toxicity of EPs, many problems remain to be addressed. For example, (1) most of the studies are under single pollutant exposure, while there are relatively few studies on co-exposure. Combined exposure can better reflect the influences of environmental pollution on the reproductive toxicity of *C. elegans*; therefore, more attention needs to be paid in the future. (2) Parent exposure obtained more attention than successive exposure. For EPs to persist in the environment, successive exposure deserves future study. (3) For mechanisms, although many potential mechanisms have been proposed, most of them are not thorough enough, only detect changes in relevant genes and speculate on possible mechanisms based on the literature. In addition, these mechanisms may not cover all aspects, as exposure to pollutants can trigger a variety of cellular responses in *C. elegans*. Therefore, more in-depth and comprehensive potential mechanisms need to be explored and presented in the future. (4) As a model organism, *C. elegans* poses several limitations which should not be omitted. *C. elegans* lacks any specific organs. When studying complex conditions, the evolutionary distance from humans might pose a significant constraint. In the future, the impact of EPs on reproduction should be comprehensively conducted in combination with *C. elegans* and other animal models. Concurrently, it is essential to emphasize the thorough assessment and mechanistic investigation of various chemical compounds to enhance our comprehension of their impacts on the physiological and genetic characteristics of living organisms. Furthermore, the development of environmental protection policies holds significant importance. Policies that are informed by scientific evidence can substantially decrease the release of environmental pollutants and mitigate harm to the ecological system.

## Figures and Tables

**Table 3:** Underlying mechanisms for reproductive toxicity of EPs on *C. elegans*.

EPs	Possible Mechanisms Involved	Genes Involved	References
PFOS/PFBS	ROS, germ cell apoptosis	*sod-3*, *ctl-2*, *gst-4*, *ced-13*, *egl-1*	[20]
DEHP	oxidative stress, DNA damage, oocyte apoptosis	*cep-1*, *ced-4*, *ced-3*, *ced-9*, *egl-1*, *mev-1*, *gas-1*	[23]
BDE-47	oxidative stress, oocyte apoptosis (p53-independent MAPK pathway)	*cep-1*, *sek-1*, *abl-1*, *mek-1*, *hus-1*, *egl-1*	[32]
BPA	StAR-mediated mitochondrial cholesterol transport	*strl-1*, *tspo-1*	[42]
TnBP	DNA damage, oxidative stress, germ cell apoptosis	*ced-4*, *ced-3*, *ced-9*, *hus-1*, *egl-1*, *clk-2*, *mev-1*, *gas-1*, *cep-1*	[31]
DEHP	autophagy, ROS	*unc-86*, *atg-7*, *atg-18*, *lgg-1*, *bec-1*, *unc-51*	[24]
PFOS/PFOA	spermatogenesis	*spe-4*, *spe-6*, *spe-10*, *spe-15*, *spe-17*, *fer-1*, *wee-1.3*, *swm-1*, *puf-8*, *try-5*	[19]
TCBPA	germ cell apoptosis, DNA damage	*cep-1*, *ced-4*, *ced-3*, *ced-9*, *hus-1*, *egl-1*, *efl-2*, *egl-38*, *lin-35*, *dpl-1*, *sir-2.1*, *pax-2*	[43]
Fosthiazate	oxidative stress, spermatogenesis	*puf-8*, *fer-1*, *swm-1*, *try-5*, *spe-5*, *wee-1.3*, *sod-3*, *sod-1*, *isp-1*, *sod-2*, *sod-5*, *gst-4*, *mlt-1*, *mlt-2*	[26]
Pristine/NPS-NH_2_	germline apoptosis, DNA damage	*cep-1*, *ced-4*, *ced-3*, *ced-9*, *hus-1*, *egl-1*, *clk-2*	[43]
Pristine/UV-PS	DNA damage, germline apoptosis	*ced-4*, *ced-3*, *ced-9*, *hus-1*, *egl-1*, *clk-2*, *cep-1*,	[37]
FNT	germ cell meiosis and sperm formation (androgen receptor signaling)	*nhr-69*, *gld-2*, *gld-3*, *fbf-1*, *fbf-2*, *fog-1*, *fog-3*	[27]
TPHP	JNK signaling pathway	*mkk-4*, *dlk-1*, *egl-15*, *jnk-1*, *vhp-1*, *kgb-2*, *kgb-1*,	[44]
ENR/CIP/NOR/OFL/FLE/LOM/SAR	oxidative stress, germ cell apoptosis	*gst-1*, *gst-4*, *mev-1*, *sod-2*, *gst-5*, *gpx-2*, *ctl-1*, *ctl-2*, *gst-3*, *gst-8*, *sod-1*, *gpx-3*, *egl-5*, *egl-38*, *pax2-b*, *dpl-1*, *ced-11*, *egl-13*, *pax3*, *ced-7*, *egl-3*	[40]
Pristine/aged PLA-MPs	DNA damage, germline apoptosis	*cep-1*, *hus-1*, *egl-1*, *clk-2*, *mrt-2*, *ced-4*, *ced-3*, *ced-9*	[38]

Abbreviations: PFOS: Perfluorooctane sulfonate; PFBS: Perfluorobutane sulfonate; DEHP: Di-(2-ethylhexyl) phthalate; BDE-47: 2,2′,4,4′-tetrabromodiphenyl ether; BPA: Bisphenol A; TnBP: Tri-n-butyl phosphate; PFOA: Perfluorooctanoic acid; TCBPA: Tetrachlorobisphenol A; NPS: Nanopolystyrene; NPS-NH_2_: Amino modified NPS; UV-PS: UV-photodegraded PS; FNT: Fenitrothion; TPHP: Triphenyl phosphate; ENR: Enrofloxacin; CIP: Ciprofloxacin; NOR: Norfloxacin; OFL: Ofloxacin; FLE: Fleroxacin; LOM: Lomefloxacin hydrochloride; SAR: Sarafloxacin hydrochloride; PLA-MPs: Polylactic acid MPs.

**Table 4:** Underlying mechanisms for multi/trans-generational reproductive toxicity of EPs on *C. elegans*.

EPs	Possible Mechanisms Involved	Genes Involved	References
DEHP	production of inadequate vitellogenin, malfunction of H3Kme2 demethylase	*vit-2*, *spr-5*, *vit-6*	[54]
HBCD	oxidative stress, cell apoptosis	*hsp-16.2*, *hsp-16.48*, *sod-1*, *sod-3*, *cep-1*	[53]
PS-NPs	epigenetic regulation	*ced-4*, *ced-3*, *ced-9*, *met-2*, *spr-5*, *set-2*	[68]
NPS/NPS-NH_2_	germ cell apoptosis	*ced-4*, *ced-3*, *ced-9*	[62]
NPS	oxidative stress	*daf-2*, *sod-3*, *mev-1*	[59]
NPS-S	germline Notch signal	*lag-2*, *glp-1*, *glb-10*, *ins-3*, *daf-28*, *ins-39*, *daf-7*, *dbl-1*	[69]
ATR	epigenetic modification (methylation of DNA and histones)	*damt-1*, *nmad-1*, *set-2*, *set-25*, *met-2*, *mes-4*, *utx-1*, *pat-12*, *wrt-3*, *flwr-1*, *lnp-1*, *fbxa-108*, *ZC317.7*, *atfs-1*, *hsp-6*, *hsp-60*	[58]
PS/PS-NH_2_/PS-COOH	histone methylation	*set-30*, *met-2*	[63]
PLA-MPs	histone methylation, secreted ligands	*ced-4*, *ced-3*, *ced-9*, *egl-1*, *cep-1*, *mrt-2*, *clk-2*, *hus-1*, *ins-39*, *wrt-3*, *met-2*, *set-6*, *mes-2*, *set-24*, *set-31*, *set-2*, *set-16*	[60]

Abbreviations: DEHP: Di-(2-ethylhexyl) phthalate; HBCD: Hexabromocyclododecane; PS: Polystyrene; PS-NPs: Polystyrene nanoplastics; NPS: Nanopolystyrene; NPS-NH_2_: Amino modified NPS; NPS-S: Sulfonate-modified NPS; ATR: Atrazine; PS-NH_2_: Amino modified PS; PS-COOH: Carboxyl modified PS; PLA-MPs: Polylactic acid MPs.

## Data Availability

Not applicable.
